# BMP4 and FGF2 promote heterogeneous priming including extraembryonic lineages in porcine expanded potential stem cells

**DOI:** 10.3389/fcell.2026.1909585

**Published:** 2026-09-09

**Authors:** Haneul Kim, Lian Cai, Sang-Hwan Hyun, Eunhye Kim

**Affiliations:** 1 Laboratory of Molecular Diagnostics and Cell Biology, College of Veterinary Medicine, Gyeongsang National University, Jinju, Republic of Korea; 2 Laboratory of Veterinary Embryology and Biotechnology, Veterinary Medical Center and College of Veterinary Medicine, Chungbuk National University, Cheongju, Republic of Korea; 3 Vet-ICT Convergence Education and Research Center (VICERC), Chungbuk National University, Cheongju, Republic of Korea

**Keywords:** BMP4, expanded potential stem cells, extraembryonic lineages, FGF2, pig

## Abstract

**Introduction:**

Extraembryonic lineages, specifically the trophectoderm (TE) and primitive endoderm (PrE), are indispensable for early embryogenesis and successful pregnancy. Expanded potential stem cells (EPSCs) can contribute to both embryonic and extraembryonic fates, providing a platform to model early lineage specification.

**Methods:**

This study investigated the effects of fibroblast growth factor 2 (FGF2) and bone morphogenetic protein 4 (BMP4) (2F condition) on lineage-associated priming in porcine EPSCs (pEPSCs).

**Results:**

Following 2F treatment, cells maintained a normal karyotype and underwent morphological changes accompanied by downregulation of pluripotency markers (*OCT4*, *SOX2*) and upregulation of TE (*CDX2* and *GATA3*) and PrE markers (*GATA4*, *GATA6*, *SOX17*, and *HNF4A*). Transcriptomic and immunofluorescence analyses consistently demonstrated activation of TE- and PrE-associated molecular programs, although markers of additional developmental lineages were also detected. These findings suggest that combined FGF2 and BMP4 treatment promotes extraembryonic lineage-associated priming in pEPSCs, and generates a heterogeneous population exhibiting features of both TE- and PrE-associated states.

**Discussion:**

Collectively, this study establishes a simple and defined *in vitro* platform for investigating signaling-dependent lineage priming and cell state remodeling in porcine EPSCs.

## 1 Introduction

Extraembryonic lineages are essential for early embryonic development and the establishment of a successful pregnancy. The primitive endoderm (PrE; also referred to as hypoblast) gives rise to the visceral and parietal endoderm of the yolk sac, which supports the embryo through nutrient transport, gas exchange, hematopoiesis, and delivery of signaling cues required for patterning until placental formation is complete (Bielinska et al., 1999; Talbot et al., 2007). As the progenitor of placenta, the trophectoderm (TE) differentiates into multiple trophoblast subtypes, such as syncytiotrophoblasts and extravillous trophoblasts, which mediate maternal–fetal interactions and facilitate nutrient transfer and gas exchange (Gamage et al., 2016; Li et al., 2019a). Despite their importance, mechanistic studies of blastocyst development remain challenging because these processes occur within the maternal environment, limiting experimental access and manipulation.

*In vitro* models that recapitulate early development require efficient derivation and stable maintenance of lineage-specific stem cells (Rivron et al., 2018; Kagawa et al., 2022; Karvas et al., 2023). Stem cell–based blastoid systems can be established from pluripotent stem cells (PSCs) alone (Li Z. et al., 2019; Kagawa et al., 2022; Zhang et al., 2023), or by co-culturing PSCs with trophoblast stem cells (TSCs) (Harrison et al., 2017; Sozen et al., 2019; Fan et al., 2021) or extraembryonic endoderm stem cells (XEN cells) (Sozen et al., 2018; Bao et al., 2022; Tu et al., 2023). These approaches enable generation of blastoids and post-implantation-like structures, such as gastruloid-like models. Therefore, robust methods for generating lineage-specific stem cells are critical for blastoid research. In pigs, a key limitation of current blastoid models is incomplete or immature extraembryonic lineage development, particularly in XEN-like cells (Xiang et al., 2024). This highlights the need for well-defined and reproducible conditions to induce extraembryonic lineage stem cells from porcine PSCs.

Signaling pathways guiding extraembryonic lineage induction have been extensively characterized in mouse and human systems. Bone morphogenetic protein 4 (BMP4) has been widely used to promote trophoblast lineage specification (Xu et al., 2002; Erb et al., 2011; Horii et al., 2019), whereas fibroblast growth factor 4 (FGF4) is commonly included in XEN derivation protocols (Frankenberg et al., 2011; Ming et al., 2024; Okubo et al., 2024). Human XEN cells have been derived from human embryonic stem cells (ESCs) using a seven-factor (7F) cocktail containing FGF4, BMP4, A83-01, XAV939, PDGF-AA, IL-6, and retinoic acid (RA) (Okubo et al., 2024). Similarly, bovine XEN cells have been generated by adapting human XEN culture conditions and applying a five-factor (5F) cocktail comprising FGF4, BMP4, A83-01, XAV939, and PDGF-AA (Ming et al., 2024). In contrast, porcine studies have largely focused on deriving XEN cells from blastocysts using FGF2 and leukemia inhibitory factor (LIF) (Li et al., 2020; Park et al., 2021; Zhang et al., 2021). More recently, culture conditions combining FGF2, LIF, and CHIR99021 have enabled the derivation of XEN-like cells from porcine ESCs (Jeong et al., 2025). Nevertheless, the overall progress in this area remains limited compared with that in mouse and human models.

Expanded potential stem cells (EPSCs) can contribute to both embryonic and extraembryonic lineages, making them a powerful platform for studying early lineage specification (Yang et al., 2017). Their unique developmental plasticity has attracted increasing interest as a means of generating extraembryonic lineage stem cells *in vitro*. However, efficient and streamlined conditions for inducing porcine extraembryonic lineages from EPSCs remain incompletely defined. In this study, we investigated whether a simplified two-factor combination of BMP4 and FGF2 could promote extraembryonic lineage-associated priming in porcine EPSCs. We found that BMP4 and FGF2 induced the emergence of cells expressing PrE- and TE-associated markers together with transcriptional changes consistent with partial activation of extraembryonic lineage-related programs. These induced populations exhibited features of heterogeneous and transitional cell states rather than fully stabilized extraembryonic stem cell identities. Collectively, our findings provide a simple *in vitro* platform for studying signaling-dependent lineage priming and cell-state remodeling in pEPSCs.

## 2 Methods

### 2.1 Mouse embryonic fibroblast (MEF) culture

MEFs were established from embryonic day 13.5 (E13.5) fetuses of Institute of Cancer Research mice and used as feeder cells. MEFs at passage 2 or 3 were inactivated with 10 μg/mL mitomycin C (M5353; Sigma-Aldrich, MO, United States) for 2–2.5 h. Inactivated MEFs were seeded at 2.5 × 10^5^ cells/mL in four-well plates coated with 0.1% gelatin (LS023-01; Welgene, Korea) and cultured in Dulbecco’s modified Eagle medium (DMEM; 11995065; Gibco, MA, United States) supplemented with 10% fetal bovine serum (FBS; Gibco), 1× GlutaMAX (Gibco), 1 × β-mercaptoethanol (Gibco), 1× MEM Non-Essential Amino Acids Solution (MEM NEAA; Gibco), and 1× antibiotic–antimycotic at 37 °C in 5% CO_2_. Antibiotic–antimycotic was initially obtained from Gibco; however, due to supply limitations, later batches were obtained from GenDEPOT (CA002-010). No differences were observed in contamination control.

### 2.2 pEPSCs culture

The pEPSCs derived from *in vitro* fertilization and parthenogenetic embryos used in this study were kindly provided by Professor Sang-Hwan Hyun (Chungbuk National University, Republic of Korea) were characterized as previously described (Cai et al., 2025) and were previously established in our laboratory. Cells were cultured on MEF feeder cells in pEPSC medium, consisting of DMEM/F12 (21331020; Gibco) and mTeSR at a 1:1 (v/v) ratio, supplemented with 0.5× N2 (Gibco), 0.5× B27 (Gibco), 0.3% FBS, 1× GlutaMAX, 1× β-mercaptoethanol, 1× MEM non-essential amino acids, 1× antibiotic–antimycotic solution, 0.2 μM CHIR99021 (4423; Tocris, UK), 0.3 μM WH-4-023 (5413; Tocris), 2.5 μM XAV939 (X3004; Sigma-Aldrich), 10 ng/mL recombinant murine LIF (250-02-25; PeproTech, Rocky Hill, NJ, United States), 20 ng/mL recombinant human/mouse Activin A (78001.1; STEMCELL Technologies, Canada), and 250 μM or 65 μg/mL L-ascorbic acid (A4544; Sigma-Aldrich). Cells were maintained at 37 °C in 5% CO_2_ with daily medium replacement. pEPSC medium was stored at 4 °C and used within 1 week. pEPSCs were dissociated using TrypLE Express Enzyme (12605-010; Gibco) and passaged every 3–4 days at a split ratio of 1:4 to 1:6 under routine culture conditions. To enhance cell survival, 10 μM ROCK inhibitor (Y-27632, 72302; STEMCELL Technologies) was added immediately upon seeding and maintained in the culture medium for initial 24 h.

### 2.3 Screening of signaling molecules for extraembryonic lineage-primed cells (2F-treated cells) induction from pEPSCs

To evaluate signaling molecules previously reported to induce human hypoblast/PrE-like identity, detached pEPSCs were seeded onto freshly prepared MEF feeder layers at a density of 2.5 × 10^5^ cells/mL and cultured in pEPSC medium supplemented with 10 μM ROCKi for 24 h.

The induction protocol was adapted from Okubo et al. (2024), and a 3-day induction period was selected in accordance with their protocol. The following day (day 0), medium was replaced with N2B27 medium composed of DMEM/F12 and Neurobasal medium at a 1:1 (v/v) ratio supplemented with 0.5× N2, 0.5× B27, 15% knockout serum replacement (KOSR), 1× GlutaMAX, 1× β-mercaptoethanol, 1× MEM non-essential amino acids, and 1× antibiotic–antimycotic solution (all from Gibco), together with seven factors (7F) [25 ng/mL FGF4 (F8424; Sigma-Aldrich), 10 ng/mL recombinant human BMP4 (314-BP-020; R&D Systems), 10 ng/mL recombinant human PDGF-AA (100-13A; PeproTech, NJ, United States), 1 µM XAV939, 3 µM A83-01 (2939; Tocris), 0.1 µM RA (R2625; Sigma-Aldrich) all added on day 0, and 10 ng/mL recombinant human IL-6, added on day 2]. In parallel, four-factor (4F) conditions consisting of 25 ng/mL FGF4, 10 ng/mL BMP4, 3 µM A83-01, and 1 µM XAV939, and two-factor (2F) conditions consisting of 25 ng/mL FGF4 and 10 ng/mL BMP4 were tested. To promote cell proliferation during the induction process, 15% KOSR was added.

FGF2, commonly used for hypoblast derivation from porcine blastocysts, was used to screen the conditions based on a culture method slightly adapted from human protocols. The 7F, 4F, and 2F conditions used for human hypoblast induction were screened by replacing FGF4 with FGF2. Unlike the human protocol, the induction medium for pEPSCs did not include Neurobasal medium. Specifically, pEPSCs were cultured in pEPSC medium supplemented with 10 μM ROCKi for 24 h and then transferred to 2F medium consisting of DMEM/F12 (Neurobasal-free) supplemented with 0.5× N2, 0.5× B27, 15% KOSR, 1× GlutaMAX, 1× β-mercaptoethanol, 1× MEM non-essential amino acids, and 1× antibiotic–antimycotic solution (all from Gibco), together with 25 ng/mL recombinant human basic FGF (FGF2; AF-100-18B; or 100-18B PeproTech) and 10 ng/mL recombinant human BMP4. Cells were cultured for 3 days at 37 °C in 5% CO_2_ with daily medium replacement. Cells subjected to this condition are referred to as “2F-treated cells.” 2F-treated cells were dissociated using TrypLE Express (12605-010; Gibco) and passaged every 3–4 days at a 1:4–1:6 ratio. After passaging, 10 μM ROCKi was added for 24 h.

### 2.4 Assessment of signaling pathway inhibitors

2F-treated cells were cultured in induction medium supplemented with 25 ng/mL FGF2 and 10 ng/mL BMP4 and treated for 3 days with either 10 µM SU5402 (3300; Tocris) or 0.5 µM LDN193189 (04-0074; Stemolecule™), or with a combination of both inhibitors. Cells were cultured for 3 days at 37 °C in 5% CO_2_ with daily medium replacement.

### 2.5 AP activity assay

AP activity was assessed using NBT/BCIP stock solution (11681451001; Roche). A working solution was prepared by diluting 20 μL stock solution in 1 mL 0.1 M Tris-HCl buffer (pH 9.5). Cells were washed with Dulbecco’s phosphate-buffered saline (dPBS) without calcium chloride and magnesium chloride (LB 001–02; Welgene) and fixed in 4% paraformaldehyde (BPP-9004; T&I, Korea) for 5 min at room temperature. Fixed cells were washed three times with dPBS and incubated with the working solution for 15 min at room temperature. After staining, cells were washed with dPBS, observed under a microscope, and imaged.

### 2.6 Karyotyping

Karyotyping with standard GTG-banding and cytogenetic analyses were performed by the Korea Research of Animal Chromosomes (www.krach.co.kr; Korea).

### 2.7 RT-qPCR

Comparative RT-qPCR was used to analyze the expression of progenitor markers in pEPSC-derived differentiated cells. All the samples were washed twice in dPBS, and total RNA was extracted from pEPSCs and derived 2F-treated cells using TRIzol reagent (15596026; Invitrogen, MA, United States) according to the manufacturer’s instructions. Isolated RNA (1 μg) was reverse-transcribed into cDNA using PrimeScript RT Master Mix (RR036A; Takara, Japan) according to the manufacturer’s instructions. RNA and cDNA were stored at −80 °C and −20 °C, respectively. RT-qPCR was performed in 20 μL reactions using 2× Power SYBR Green Master Mix (A25776; Applied Biosystems, MA, United States) and 10 pmol of each primer (Macrogen, Korea) (Supplementary Table S1). The reactions were performed for 40 cycles under the following conditions: denaturation at 95 °C for 15 s and annealing and extension at 57 °C for 1 min. Relative quantification was based on the Ct method, wherein Ct corresponds to the cycle in which PCR product fluorescence exceeds a defined threshold during the exponential amplification phase. The relative expression (R) was calculated using equation *R* = 2 *− (Ct*_*sample*_ *− Ct*_*control*_ *)*. The R-values for each gene were normalized to *GAPDH* expression. All experiments were performed at least three times.

### 2.8 Bulk RNA-seq and data analysis

Total RNA was extracted from pEPSCs and 2F-treated cells using TRIzol reagent (Invitrogen). cDNA library preparation and sequencing were performed by Theragen Etex (Korea; www.theragenetex.com). Libraries were generated using the TruSeq Stranded mRNA Sample Preparation Kit (Illumina, CA, United States). Polyadenylated mRNA was purified from 1 μg total RNA using oligo (dT) magnetic beads and fragmented. Random hexamer primers were used to synthesize single-stranded cDNAs from the fragmented mRNAs. Next, second-strand synthesis was performed using single-stranded cDNAs as templates, resulting in double-stranded cDNA. End repair, A-tailing, adaptor ligation, and PCR amplification were performed according to the manufacturer’s protocol. Library quality was assessed using the Agilent 2100 BioAnalyzer (Agilent, CA, United States), and libraries were quantified using the KAPA Library Quantification Kit (Kapa Biosystems, MA, United States). Sequencing was performed on an Illumina NovaSeq X Plus platform as paired-end 2 × 150 bp reads. Reads with >10% n bases, >40% bases with a Phred score of <20, or an average Phred score of <20 were considered to be of low quality and filtered using in-house scripts. Filtered reads were mapped to the species reference genome using STAR v2.4.0b (Dobin et al., 2013). Gene expression levels were quantified using Cufflinks v2.1.1 with species-specific gene annotations (Trapnell et al., 2010). Non-coding regions were masked using the--mask-file option, and the--multi-read-correct and--frag-bias-correct options were applied to improve quantification accuracy. All other options were set to the default values. Differentially expressed genes (DEGs) were identified using the TCC package in R (Sun et al., 2013). Gene-level count data were generated using HTSeq-count v0.6.1p1 (Anders et al., 2015), using the options -m intersection-nonempty, and -r for paired-end reads. The normalization factors were estimated using the iterative DEGES/edgeR method. Q-values were computed from p-values using the p. adjust function in R with default parameters. Genes with q < 0.05 were considered significant after multiple testing (Benjamini and Hochberg, 1995). GO annotation (biological process, cellular component, and molecular function) and enrichment analysis were performed using Fisher’s exact test (Fisher, 1922), and GO terms with p < 0.001 were considered statistically significant.

### 2.9 Immunofluorescence staining

For 2D cultures, cells were washed with dPBS (LB 001–02; Welgene) and fixed in 4% paraformaldehyde (Electron Microscopy Sciences) for 5 min at room temperature. Cells were washed with dPBS and permeabilized in blocking buffer consisting of dPBS, 0.5% Triton X-100, 5% donkey serum, and 0.1% bovine serum albumin for 1 h at room temperature. Cells were incubated with primary antibodies diluted in blocking buffer at 4 °C overnight (Supplementary Table S2). The next day, samples were washed three times with wash buffer (dPBS, 0.2% Tween-20) and incubated with secondary antibodies for 1 h at room temperature, and washed with wash buffer three times for 5 min each. Nuclei were stained with ′,6-diamidino-2-phenylindole (DAPI; D8417, Sigma-Aldrich) for 2 min, washed with dPBS, and mounted using VECTASHIELD (Vector Laboratories, CA, United States of America). The stained cells were observed under a microscope and imaged using software.

### 2.10 Statistical analysis

Statistical analyses were performed using GraphPad Prism (version 8.4.3). Comparisons among groups were conducted using one-way analysis of variance (ANOVA). Data are presented as mean ± SEM. Statistical significance was defined as p < 0.05. Details regarding the statistical tests, exact p-values, and biological replicates are provided in the figure legends.

## 3 Results

### 3.1 Exploring signaling conditions that induce extraembryonic lineage-associated priming in pEPSCs

**FIGURE 1:**
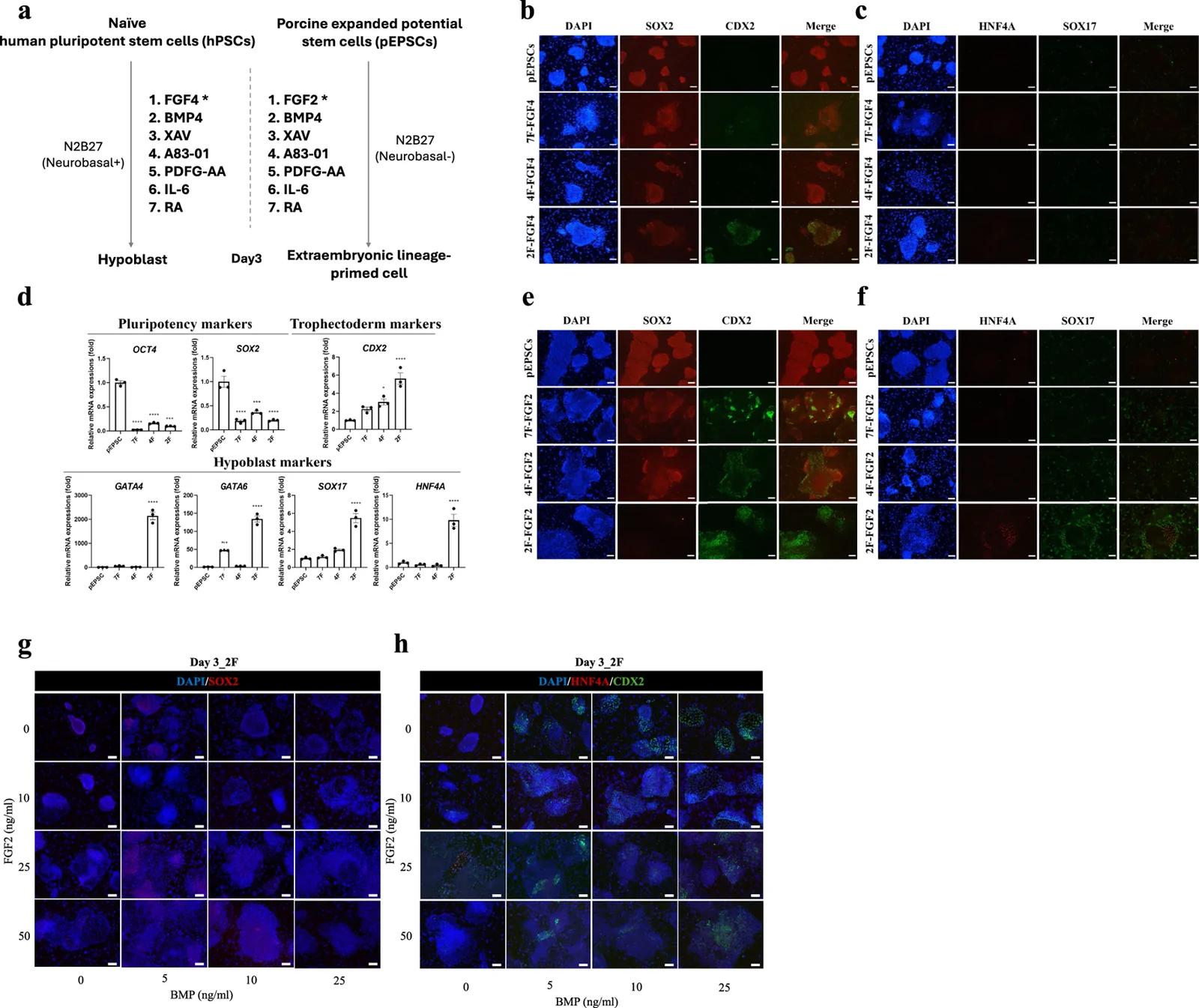
Screening of FGF/BMP4 signaling conditions for extraembryonic lineage-associated priming in pEPSCs. **(a)** Schematic overview of the experimental workflow. Asterisk indicates FGF ligand used (FGF4 for hPSCs; FGF2 for pEPSCs). 7F, 7-factor condition; 4F, 4-factor condition; 2F, 2-factor condition. **(b)** Immunofluorescence analysis of pluripotency (SOX2) and trophectoderm (CDX2) markers in pEPSCs under FGF4-based 7F, 4F, or 2F conditions. Scale bar, 100 μm. **(c)** Immunofluorescence analysis of primitive endoderm markers (HNF4A and SOX17) in pEPSCs under FGF4-based 7F, 4F, or 2F conditions. Scale bar, 100 μm. **(d)** Relative expression levels of pluripotency (*OCT4* and *SOX2*), primitive endoderm (PrE; *GATA4, GATA6, SOX17,* and *HNF4A*), and trophectoderm (TE; *CDX2*) markers. Data are presented as mean ± SEM (n = 3). P values were calculated using one-way ANOVA. *p < 0.05, ***p < 0.001, ****p < 0.0001. Experiments were repeated at least three times. **(e)**Immunofluorescence analysis of pluripotency (SOX2) and TE (CDX2) markers in pEPSCs under FGF2-based 7F, 4F, or 2F conditions. Scale bar, 100 μm. **(f)** Immunofluorescence analysis of PrE markers (HNF4A and SOX17) in pEPSCs under FGF2-based 7F, 4F, or 2F conditions. Scale bar, 100 μm. **(g)** Immunofluorescence images showing SOX2 expression (red) counterstained with DAPI (blue) in pEPSCs treated with increasing concentrations of FGF2 and BMP4. Scale bars, 100 μm. **(h)** Immunofluorescence images showing HNF4A (red) and CDX2 (green) with DAPI (blue) in pEPSCs treated with increasing concentrations of FGF2 and BMP4. Scale bars, 100 μm.

To determine whether pEPSCs can be induced toward extraembryonic lineage-associated states, we first applied a chemical cocktail containing FGF4, which was previously used to induce XEN cells from human ESCs (Okubo et al., 2024). These human XEN induction conditions (7F, 4F, and 2F) were originally established using naïve human ESCs, which are permissive for hypoblast/XEN induction. Because the pEPSCs used in this study were previously characterized by comparative transcriptomic analyses as corresponding to a pluripotent state relatively close to the naïve state within the formative continuum (Cai et al., 2025), we evaluated whether these induction conditions could similarly promote extraembryonic lineage-associated priming in porcine EPSCs. Accordingly, pEPSCs were cultured under the 7F, 4F, or 2F conditions for 3 days (Figure 1a). Immunofluorescence analysis showed that pEPSCs (control) expressed the pluripotency marker SOX2 but not the TE marker CDX2 (Figure 1b) or PrE markers (HNF4A and SOX17; Figure 1c). Similar marker expression profiles were observed under 7F and 4F conditions. In contrast, under 2F (FGF4 and BMP4) conditions, SOX2 expression was maintained, and CDX2 expression was detected (Figure 1b), whereas PrE markers remained undetectable (Figure 1c).

Given that the limited induction of PrE markers with FGF4 could potentially be associated with species-specific differences in ligand activity, pairwise protein sequence alignments (NCBI BLASTp) were performed between human and porcine orthologs of FGF2, FGF4, and BMP4. FGF4 showed comparatively lower identity (92.23%), whereas FGF2 and BMP4 showed high sequence identity between human and pig (98.71% and 98.29%, respectively) (Supplementary Table S3). Based on previous reports that FGF2 supports the derivation and maintenance of porcine XEN cells from blastocysts (Li et al., 2020; Park et al., 2021; Zhang et al., 2021), FGF4 was replaced with FGF2, and the resulting conditions were re-evaluated. RT-qPCR analysis showed that the pluripotency markers *OCT4* and *SOX2* were significantly downregulated under all conditions (7F, 4F, and 2F). However, mRNA expression levels of the other lineage markers differed across conditions: 7F condition significantly upregulated the PrE marker *GATA6* (*p* < 0.001), whereas 4F condition upregulated the TE marker *CDX2* (*p* < 0.05). Moreover, 2F condition resulted in a significant upregulation of the TE marker *CDX2* and PrE markers *GATA4*, *GATA6*, *HNF4A*, and *SOX17* (*p* < 0.0001) (Figure 1d). Consistent with these results, immunofluorescence analysis showed that SOX2 expression remained unchanged under the 7F and 4F conditions, and that TE marker CDX2 expression was also detected. In contrast, under the 2F condition, CDX2 expression was maintained while SOX2 was markedly repressed (Figure 1e). SOX17 and HNF4A were not detected in the 7F and 4F conditions but were clearly induced under the 2F condition (Figure 1f). To further validate the robustness of the FGF2-based induction system, we assessed whether the previously used ligand concentrations were appropriate for supporting lineage specification under the new FGF2 + BMP4 (2F) conditions (Figures 1g,h; Supplementary Figure S1). Quantification was performed on fields randomly selected across the entire imaging area. FGF2 (25 ng/mL) in combination with BMP4 (10 ng/mL) showed a trend toward higher expression of the PrE-specific marker HNF4A (10.20% ± 8.26%), and additional representative images confirmed robust CDX2 expression (up to 88.46%, Supplementary Figure S2e, S3e). Despite the residual SOX2 (42.00% ± 30.82%) expression, the high induction of extraembryonic lineage markers led us to select this condition for subsequent experiments.

### 3.2 Induction of PrE- and TE-associated lineage priming by combined FGF2 and BMP4 condition

**FIGURE 2:**
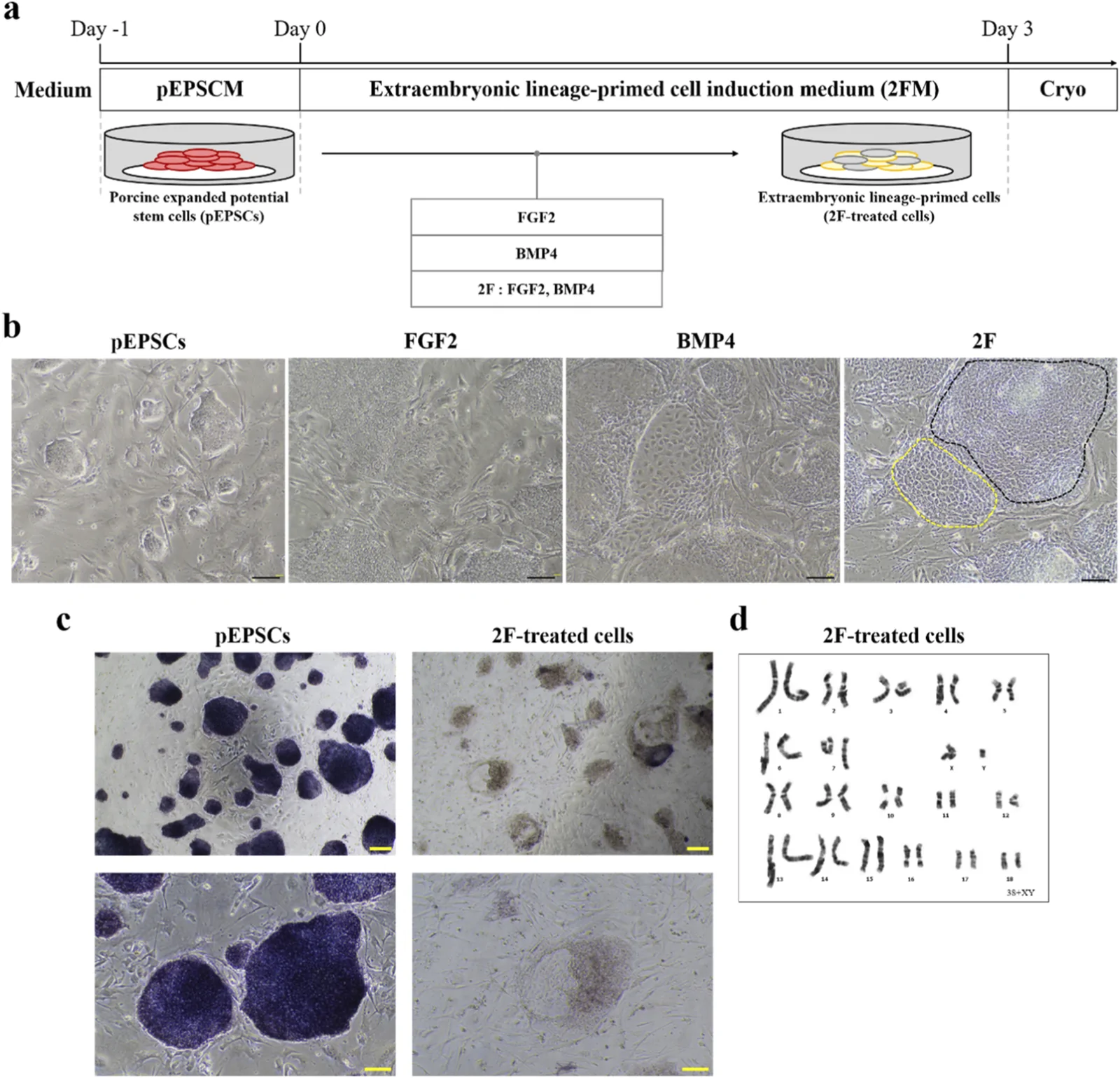
Induction and analysis of lineage-associated priming from pEPSCs under FGF2 and BMP4 conditions. **(a)** Schematic diagram of the experimental workflow for 2F-induced lineage-associated priming in pEPSCs. **(b)** Morphology of lineage-associated priming cells treated with FGF2, BMP4, and 2F. Scale bar, 100 μm. Black dotted line: compact colony; yellow dotted line: non-compact colony. **(c)** Alkaline phosphatase activity in pEPSCs and 2F-treated cells. Scale bar: 200 μm (top), 100 μm (bottom). **(d)** Karyotyping analysis of 2F-treated cells (38+XY).

Cells induced under the FGF2 + BMP4 (2F) condition were further characterized using morphological and molecular assays (Figure 2a). Control pEPSCs formed dome-shaped, tightly packed colonies (Figure 2b). In contrast, cells treated with FGF2 or BMP4 alone exhibited flattened colony morphologies, with BMP4-treated cells frequently forming loosely organized colonies. Under the 2F condition, colonies showed a flattened morphology and could be further categorized into compact and non-compact colony types. Alkaline phosphatase (AP) staining revealed strong AP activity in control pEPSCs, whereas 2F-treated cells showed no detectable AP activity (Figure 2c), consistent with loss of pluripotent characteristics. Karyotype analysis demonstrated that 2F-treated cells maintained a normal chromosomal complement (Figure 2d), indicating cytogenetic stability following induction.

**FIGURE 3:**
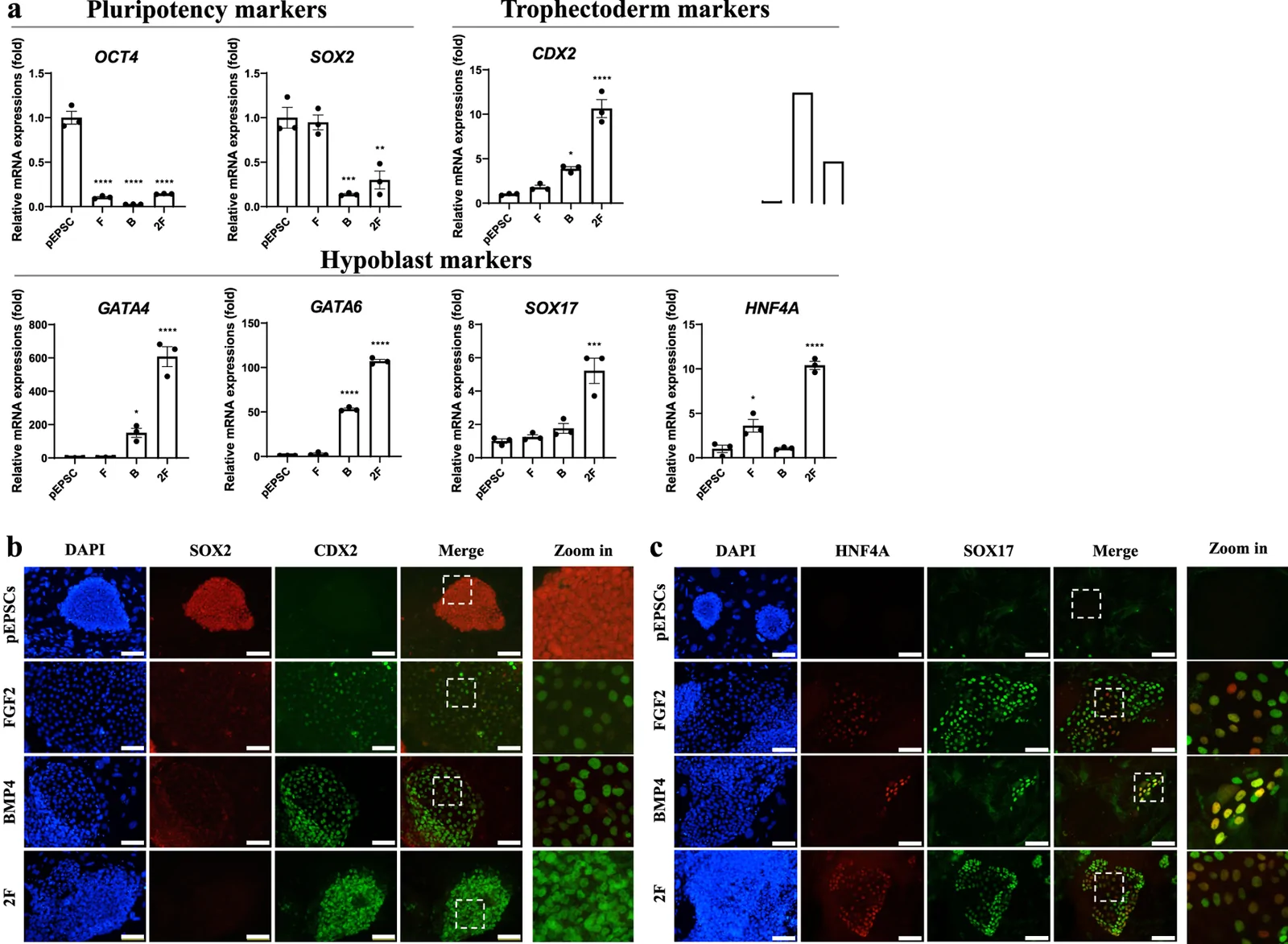
Expression of lineage-specific markers in the extraembryonic lineage-primed cells derived from pEPSCs. **(a)** RT-qPCR analysis of the expression of pluripotency (*OCT4* and *SOX2*), TE (*CDX2* and *GATA3*), and PrE (*GATA4*, *GATA6*, *SOX17*, and *HNF4A*) markers in 2F-treated cells. F, FGF2; B, BMP4; 2F, combined FGF2 and BMP4. Data represent the mean ± SEM(n = 3). mRNA expression levels were quantified by qPCR and normalized to GAPDH. The *p*-values were calculated using one-way ANOVA. *p < 0.05, **p < 0.01, ***p < 0.001, ****p < 0.0001. **(b)** Immunofluorescence analysis of pluripotency (SOX2) and TE (CDX2) markers in 2F-treated cells on day 3. Scale bar, 100 μm. The white dashed box indicates the magnified region. **(c)** Immunofluorescence analysis of PrE markers (HNF4A and SOX17) in 2F-treated cells on day 3. Scale bar, 100 μm. The white dashed box indicates the magnified region.

**FIGURE 4:**
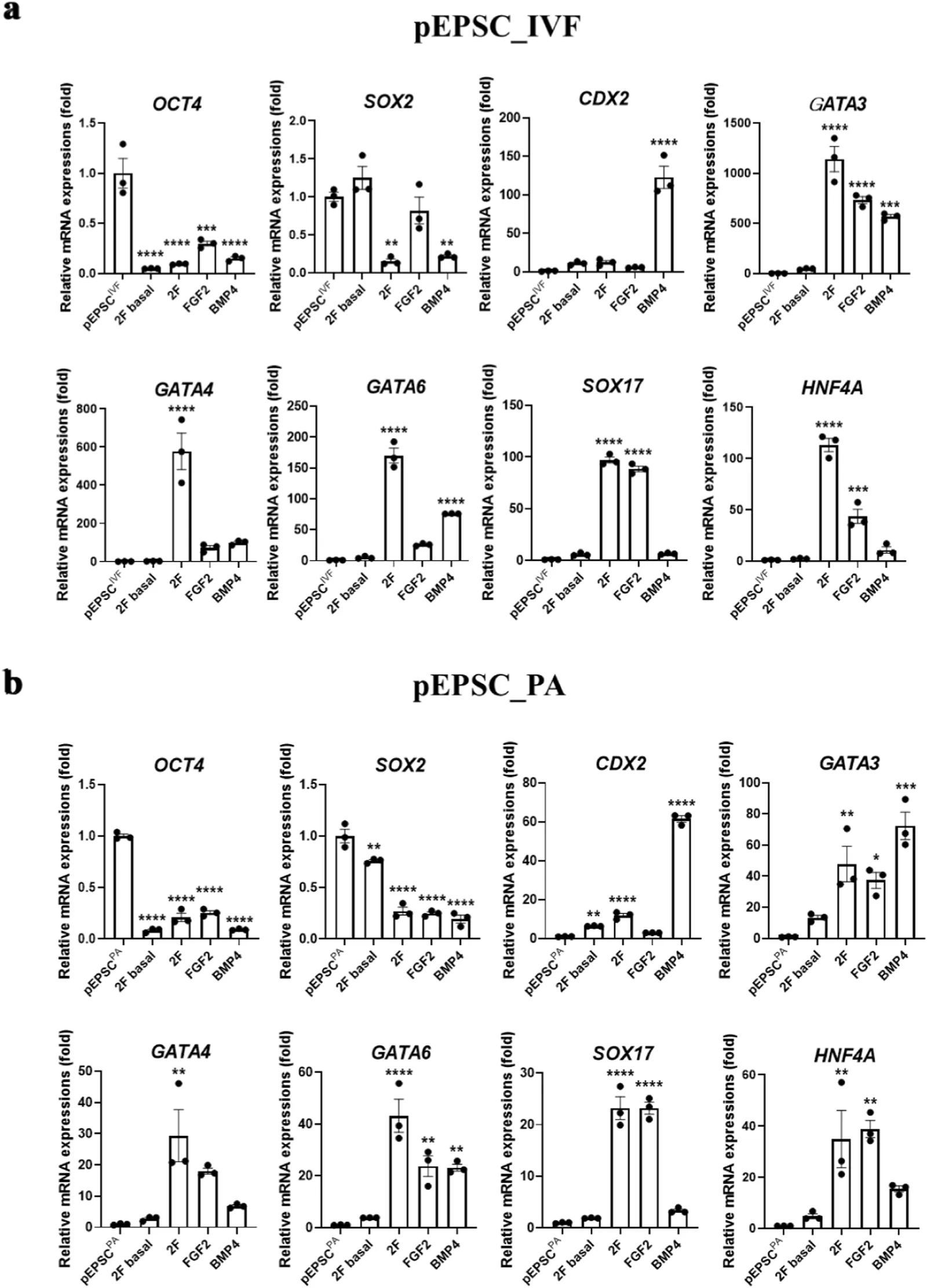
Validation of gene expression patterns in pEPSC^IVF^ and EPSC^PA^ line by qPCR. **(a)** qPCR analysis of gene expression in the pEPSC^IVF^ line. **(b)** qPCR analysis of gene expression in the pEPSC^PA^ line. *P*-values were calculated using one-way ANOVA. *p < 0.05, **p < 0.01, ***p < 0.001, ****p < 0.0001. Data are presented as the mean ± SEM (n = 3). Gene expression was quantified using qPCR and normalized to GAPDH.

To dissect the individual contributions of FGF2 and BMP4, lineage marker expression was assessed using RT-qPCR after exposure to FGF2 alone, BMP4 alone, or both factors (2F) (Figure 3a). *OCT4* was significantly downregulated under all treatment conditions (*p* ≤ 0.0001). However, *SOX2* was significantly downregulated under BMP4 alone (*p* < 0.001) and 2F (*p* < 0.01) treatments, but not under FGF2 alone treatment. BMP4 treatment significantly upregulated *CDX2* (*p* < 0.05) and *GATA3* (*p* < 0.0001), which were further enhanced with the 2F treatment (*p* < 0.0001 for both). With respect to PrE markers, FGF2 treatment alone significantly increased *HNF4A* expression (*p* < 0.05), with no significant effect on the expression of *GATA4*, *GATA6*, and *SOX17*. BMP4 alone treatment significantly upregulated *GATA4* (*p* < 0.05) and *GATA6* (*p* < 0.0001), whereas 2F treatment resulted in stronger upregulation of all PrE markers, including *GATA4*, *GATA6*, *HNF4A* (*p* < 0.0001 for all), and *SOX17* (*p* < 0.001). Immunofluorescence analysis further supported these findings. Under FGF2 or BMP4 alone conditions, cells expressed both CDX2 and SOX2, with a subset of cells co-expressing both markers within the same colonies (Figure 3b; Supplementary Figure S4). In contrast, under 2F conditions, no such co-expression was observed, and SOX2 was absent in regions where CDX2 was expressed. The expression of PrE marker HNF4A was detected in a subset of cells treated with both FGF2-alone and BMP4-alone conditions (Figure 3c), whereas SOX17 expression was observed in broadly non-compact cells under FGF2 alone conditions and in a subset of cells under BMP4 alone conditions. All PrE markers were detected under 2F conditions. To assess whether the observed effect was reproducible across different pEPSC lines, we performed the same analysis in pEPSCs derived from parthenogenetic embryos and observed broadly similar trends in lineage marker expression compared to IVF-derived pEPSCs (Figures 4a,b), supporting the generalizability of 2F-induced lineage priming across pEPSC lines of different origins.

**FIGURE 5:**
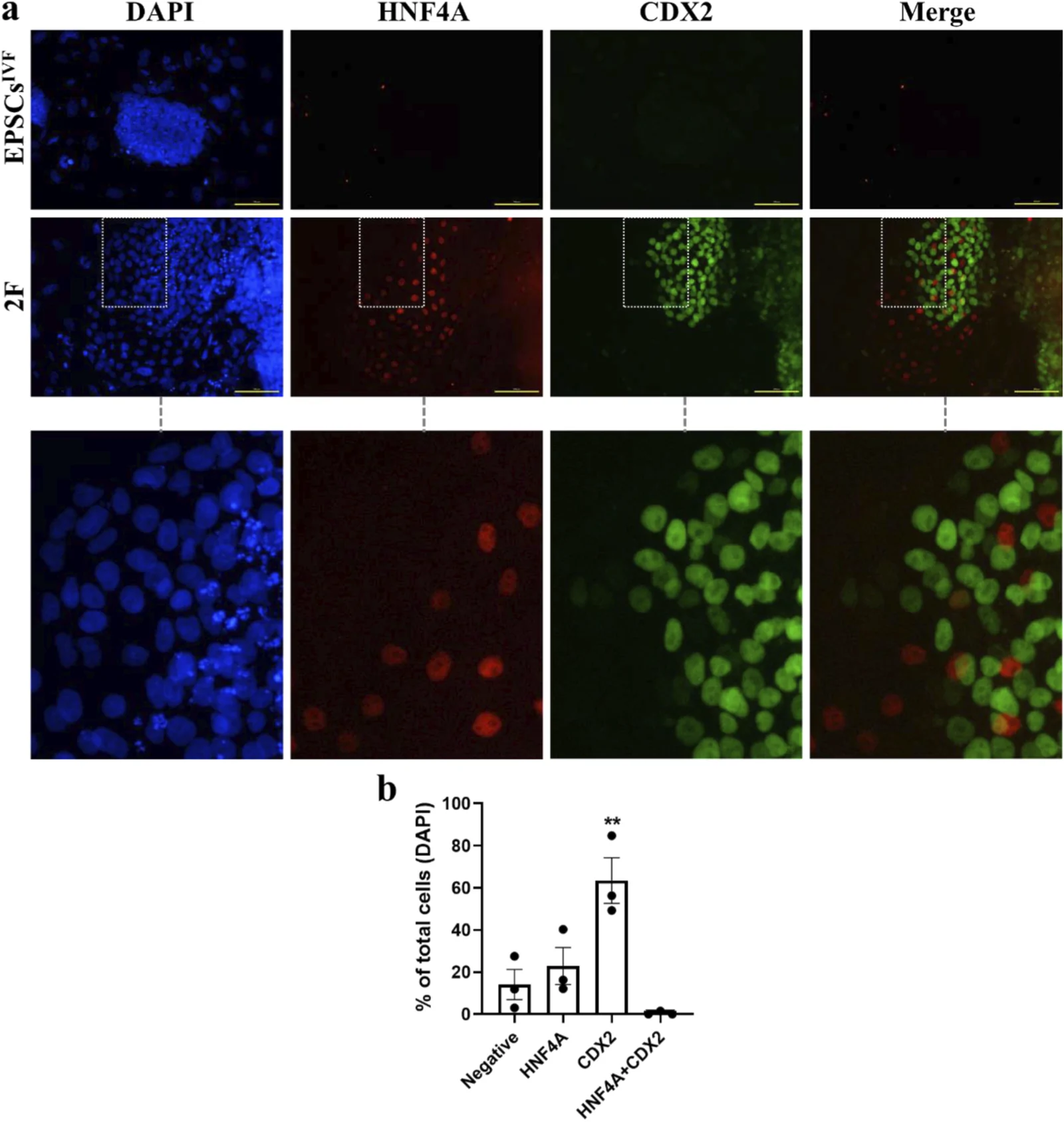
Lineage-specific expression and quantification of TE and PrE markers co-expression under 2F conditions. **(a)** Immunofluorescence analysis of primitive endoderm (HNF4A) and trophectoderm (CDX2) markers. Scale bar, 100 μm. The white dashed box indicates the magnified region. **(b)** Quantification of marker-positive and co-expressing cells (%). Quantification was performed from three independent microscopic fields of the same sample and is presented as mean ± SEM. CDX2^+^ (50.1% ± 21.0%) and HNF4A^+^ (36.2% ± 13.2%) cells were detected. Double-positive cells were rare (0.5% ± 0.5%), and 14.1% ± 7.2% were negative.

**FIGURE 6:**
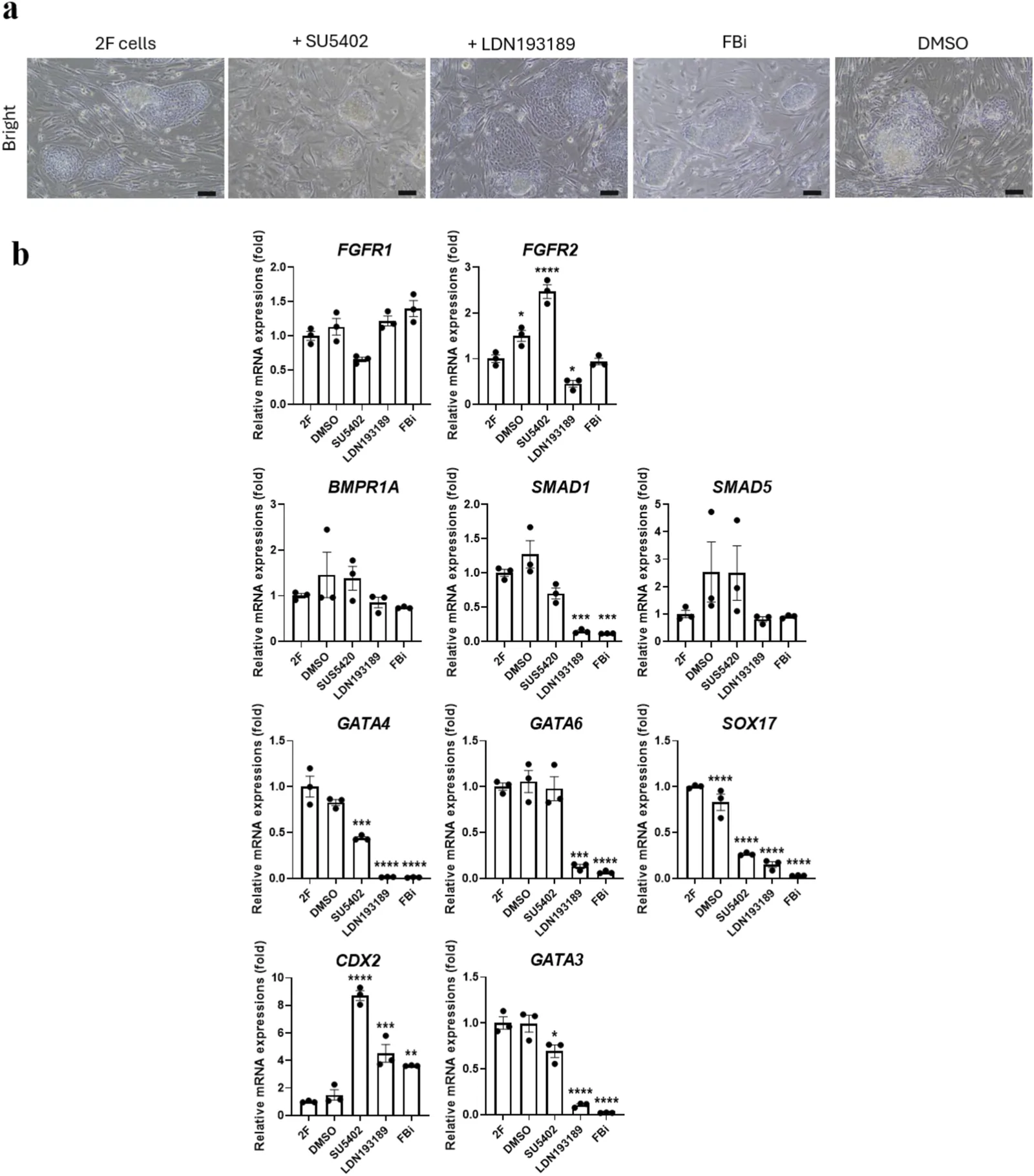
Analysis of signaling pathway dependence in 2F-treated cells. **(a)** Morphology of 2F-treated cells cultured in 2F, 2F + DMSO, 2F + 10 μM SU5402, 2F + 0.5 μM LDN193189, 2F + 10 μM SU5402, and 0.5 μM LDN193189 (FBi). 10 inhibitor conditions. Scale bar, 100 μm. **(b)** RT-qPCR analysis of FGF (*FGF receptor1* and *2*), BMP (*BMP receptor, SMAD1, SMAD5*) signaling and PrE (*GATA4, GATA6. SOX17*) and TE (*CDX2, GATA3*) markers. *p < 0.05, **p < 0.01, ***p < 0.001, ****p < 0.0001. Data represent the mean ± SEM (n = 3). qPCR quantification of gene expression normalized to GAPDH.

We next analyzed the co-expression of lineage-specific markers to determine whether 2F-treated cells exist in a bipotential state (Figure 5). Immunofluorescence analysis revealed that the TE and PrE markers (CDX2 and HNF4A, respectively) were expressed in mutually exclusive cell populations, suggesting divergence toward distinct lineage-associated states rather than a uniformly bipotential population. Next, we performed loss-of-function experiments using the FGF receptor inhibitor SU5402 and the BMP type I receptor inhibitor LDN193189 to investigate the respective and synergistic roles of FGF and BMP signaling under 2F conditions (Figures 6a,b). SU5402 resulted in reduced cell proliferation and a moderate decrease in the expression of PrE-associated markers, such as GATA4 and SOX17, suggesting that FGF signaling contributes to both proliferation and PrE-associated gene expression. In contrast, LDN193189 did not substantially affect proliferation but led to a pronounced downregulation of PrE markers, including GATA4, GATA6, and SOX17, indicating a broader role for BMP signaling in regulating PrE-like gene expression. Notably, TE-associated markers such as CDX2 and GATA3 were also reduced upon BMP inhibition, suggesting that BMP signaling broadly contributes to lineage-associated transcriptional programs under 2F conditions. Collectively, these results suggest that both FGF and BMP signaling contribute to extraembryonic lineage-associated priming, with BMP signaling exerting a broader influence on both PrE- and TE-associated transcriptional programs.

### 3.3 Transcriptomic profiling of 2F-treated cells derived from pEPSCs

**FIGURE 7:**
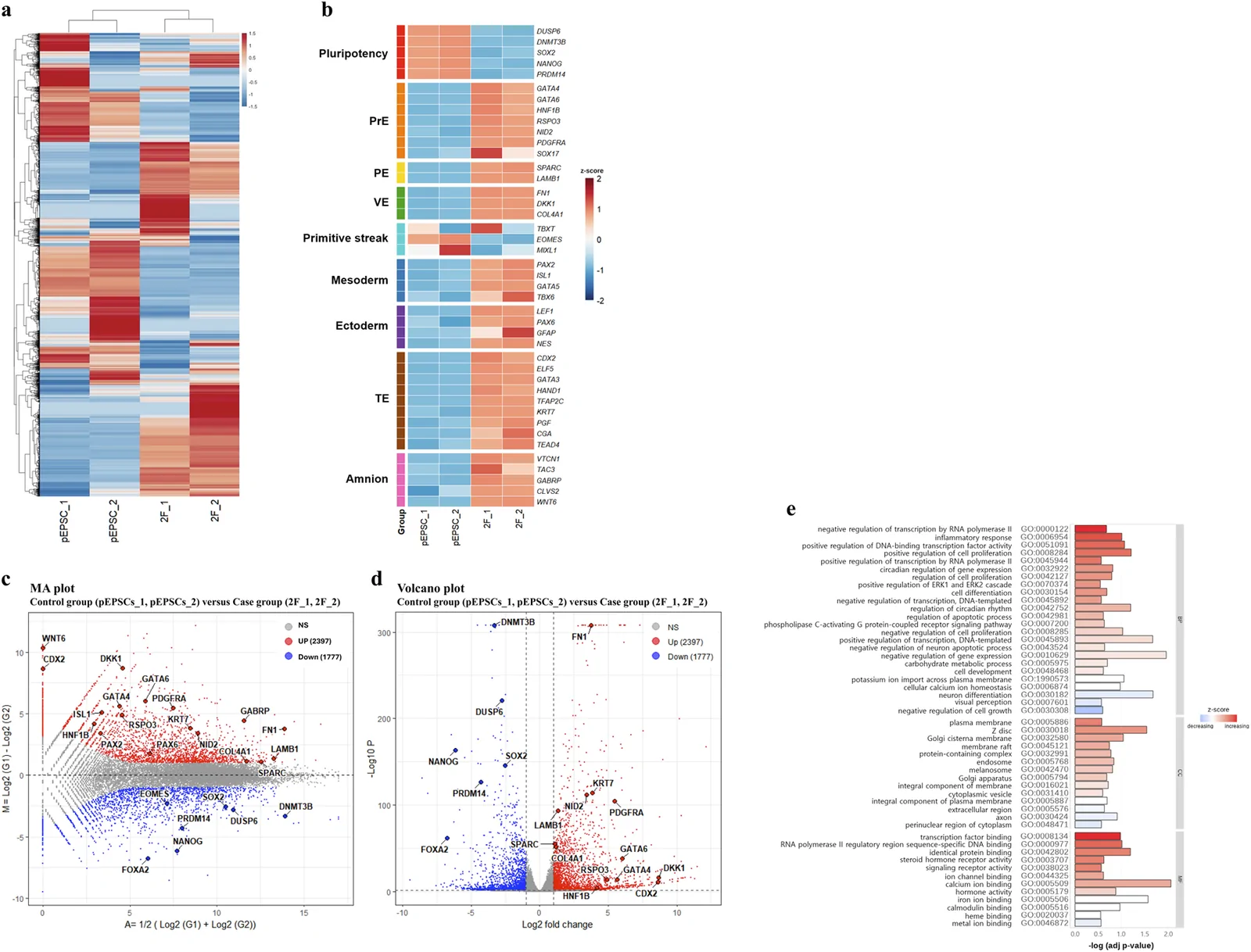
Expression heatmap, MA plot, and volcano plot of bulk RNA-seq data comparing pEPSCs and 2F-treated cells. **(a)** Global gene heatmap for pEPSCs and 2F-treated cells. **(b)** Heatmap of lineage-specific gene expression in pEPSCs and 2F-treated cells. Gene expression values (TPM) were log2-transformed [log2 (TPM+1)] and subsequently scaled using gene-wise z-score normalization (mean-centered and divided by standard deviation across samples). **(c)** MA plot showing the relationship between average gene expression and log_2_ fold change between pEPSCs and 2F-treated cells. **(d)** Volcano plot showing differentially expressed genes between pEPSCs and 2F-treated cells. Significantly upregulated and downregulated genes in 2F-treated cells are shown in red and blue, respectively, and non-significant genes are shown in gray based on fold change and statistical significance. **(e)** Gene ontology analysis comparing pEPSCs and 2F-treated cells. GoBar representing enriched GO terms in the molecular functions, biological processes, and cellular components. Blue, downregulated; Red, upregulated. n = 2 biological replicates for both pEPSCs and 2F-treated cells.

To further characterize the transcriptional changes induced by the 2F condition, bulk RNA sequencing (RNA-seq) was performed to compare 2F-treated cells and control pEPSCs (Figure 7a). Global gene expression heatmap analysis revealed that 2F-treated cells exhibited transcriptional profiles distinct from those of pEPSCs, indicating a change in cellular identity. Lineage-specific marker analysis showed induction of both PrE *(GATA4, GATA6, SOX17, PDGFRa*, and *HNF1B)* and TE (*CDX2, ELF5, GATA3, HAND1, TFAP2C, KRT7*, *PGF, CGA,* and *TEAD4*) lineage markers in 2F-treated cells, accompanied by downregulation of pluripotency-related markers (*NANOG*, *SOX2*, and *PRDM14*) and the pEPSC-associated marker *DUSP6* (Figure 7b) (Malik et al., 2022). Moreover, 2F-treated cells expressed both parietal and visceral markers, suggesting that the PrE-like population within these cells is associated with yolk sac-related transcriptional programs, including both the parietal and visceral endoderm. Additional developmental lineage markers were also detected, including mesoderm lineage markers (*PAX2, ISL1, GATA5*, and *TBX6*) and ectoderm lineage markers (*LEF1, PAX6, GFAP,* and *NES*) and amnion lineage markers (*VTCN1, TAC3, GABRP, CLVS2,* and *WNT6*). Primitive streak markers (*EOMES* and *MIXL1*) were relatively higher in pEPSCs compared with the 2F-treated cells. In contrast, *TBXT* did not show a consistent pattern between pEPSCs and 2F-treated cells. To validate this observation, RT-qPCR was performed for *TBXT* (Supplementary Figure S5). In addition, *OCT4* (pluripotency), *ISL1* (mesoderm) and *PAX6* (ectoderm) were validated by RT-qPCR. *OCT4* was significantly decreased, and *ISL1* significantly increased. *TBXT* and *PAX6* exhibited a tendency toward upregulation in the 2F treated cell, but the difference was not statistically significant (*p* = 0.082 and *p* = 0.598 respectively). Overall consistent with the bulk RNA-seq data. Collectively, the transcriptomic profile suggests that 2F-treated cells acquire lineage-associated features characterized by activation of PrE- and TE-related programs together with broader developmental plasticity. Furthermore, differential expression analysis identified 2,397 upregulated and 1,777 downregulated genes among the 35,682 transcripts in 2F-treated cells (Figure 7c). Consistent with the heterogeneous lineage-associated markers, volcano plot analysis further demonstrated significant downregulation of pluripotency- and pEPSC-related genes (Malik et al., 2022), such as *NANOG*, *SOX2*, *PRDM14*, *DUSP6*, and *DNMT3B* and upregulation of multiple lineage-associated genes, including TE and PrE genes (*CDX2*, *GATA4*, *GATA6*, *DKK1*, *HNF1B*, *PDGFRA*, and *FN1*), mesoderm (*PAX2, ISL1, GATA5, TBX6*) and ectoderm (*PAX6, NES*) genes (Figure 7d). These findings indicate that 2F treatment drives a transcriptome-wide transition from a pluripotent state to broad transcriptional remodeling, rather than uniform specification toward a single lineage identity.

To further examine the functional context of these transcriptional changes, Gene Ontology (GO) enrichment analysis was performed (Figure 7e). The enriched terms were associated with transcriptional regulation, cell proliferation, and membrane-associated processes, suggesting broad changes in cellular identity and behavior following 2F treatment.

## 4 Discussion

Early blastocyst development depends on tightly coordinated interactions between the embryonic and extraembryonic lineages (Stephenson et al., 2012; Okubo et al., 2024). Understanding these interactions is essential for elucidating the fundamental principles underlying mammalian development. Recapitulating early lineage specification *in vitro* requires not only epiblast-derived stem cells but also tractable models representing extraembryonic lineages, including PrE and TE. Extraembryonic cells play indispensable roles in implantation and provide critical cues that guide epiblast development (Okubo et al., 2024). Although pigs are increasingly recognized as valuable preclinical large-animal models (Lunney et al., 2021), experimentally accessible systems for studying porcine extraembryonic lineage specification remain comparatively underexplored relative to mouse and human models. In this study, we show that a simplified 2F condition comprising FGF2 and BMP4 promotes extraembryonic lineage-associated priming in pEPSCs through activation of PrE- and TE-associated molecular programs. To our knowledge, this study provides one of the first demonstrations that defined signaling cues can induce broad extraembryonic lineage-associated transcriptional remodeling in porcine EPSCs under a simple culture condition.

FGF signaling is a central regulator of epiblast and PrE segregation within the inner cell mass (ICM) during early mammalian development. In mice, FGF4 promotes PrE differentiation, and its loss impairs PrE formation (Feldman et al., 1995), whereas high levels of exogenous FGF4 can convert the entire ICM into PrE (Yamanaka et al., 2010). Consistent with these observations, FGF4-containing cocktails have been used to derive XEN-like cells from human ESCs (hESCs) (Okubo et al., 2024) and bovine EPSCs (Ming et al., 2024). However, in the present study, FGF4-based conditions were not effective in inducing PrE-associated markers in pEPSCs, suggesting species-specific differences in response to FGF signaling. Additionally, sequence conservation between human and porcine orthologs was lower for FGF4 than for FGF2. Although the functional consequences of the amino acid sequence differences between porcine and human FGF4 on biological activity remain unclarified, species-specific differences in a small number of receptor contact residues have been reported to determine ligand-receptor compatibility (Gow et al., 2012). Therefore, sequence divergence between human and porcine FGF4 may have similarly influenced receptor binding or downstream signaling efficiency in pEPSCs, although this requires further validation. FGF2 has been reported to induce hypoblast-like differentiation in two-dimensional (2D) cultures and blastoids in human naive PSCs (Dattani et al., 2024). It has also been successfully used to derive XEN cells from porcine blastocysts (Shen et al., 2019; Li et al., 2020; Park et al., 2021; Zhang et al., 2021). Together, these findings suggest that FGF2 may be a more appropriate FGF ligand than FGF4 for activating PrE-related programs in porcine cells, particularly when combined with additional inductive cues.

BMP4 is a well-established inducer of trophoblast differentiation in mouse and human models (Xu et al., 2002; Bernardo et al., 2011). It has also been used as a component of chemical cocktails for the derivation of TE-like cells in human and pig models (Soncin et al., 2022; Kim et al., 2024). In hESCs, BMP4 treatment alone leads to the downregulation of pluripotency markers, such as *OCT4* and *SOX2*, and the upregulation of TE markers, such as *CDX2* and *KRT7* (Marchand et al., 2011). Consistent with these findings, BMP4 treatment of pEPSCs reduced the expression of pluripotency markers while increasing that of TE-related markers, suggesting that BMP4 similarly promotes TE-lineage priming in pigs. Consistently, transcriptomic analysis in the present study revealed the upregulation of TE-associated genes, including *CDX2, ELF5, GATA3, HAND1, TFAP2C, KRT7, PGF, CGA*, and *TEAD4*, in 2F-treated cells. Among these, *CDX2* and *TEAD4* are widely recognized as early trophectoderm markers involved in establishing TE identity and transcriptional regulation during early blastocyst development (Strumpf et al., 2005; Nishioka et al., 2008; Bernardo et al., 2011), *KRT7* is a pan-trophoblast marker (Kojima et al., 2017), and *GCM1* and *CGA* are late trophoblast markers (Gao et al., 2019). Overall, the TE-associated gene expression profile observed in 2F-treated cells substantially overlapped with the trophoblast-associated transcriptional program reported by Gao et al. (2019). Notably, however, *GCM1* (a key regulator of syncytiotrophoblast (STB) formation) was undetectable in either pEPSCs or the 2F condition, suggesting that although 2F treatment activates TE-associated molecular programs similar to those described by Gao et al. (2019), it does not fully recapitulate the trophoblast state reported in that study and is more consistent with an early TE-lineage-associated priming state. Interestingly, transcriptomic analysis revealed a concurrent upregulation of amnion-specific markers (*VTCN1*, *TAC3*, *GABRP*, *CLVS2*, and *WNT6*) in 2F-treated cells. Zheng et al. (2022) suggested that BMP4-treated primed hPSCs resemble amniotic ectoderm, unlike naïve hPSC-derived trophoblast-like cells. Considering this, the co-activation of TE- and amnion-associated markers in 2F condition likely reflects a broader, heterogeneous lineage-priming response driven by BMP4 signaling, rather than an exclusive transition toward a single lineage. Notably, the heterogeneous distribution of cells within colonies introduces variability in image-based quantification. Apparent discrepancies between mRNA and protein-level marker expression (e.g., SOX2, HNF4A, and CDX2) are more likely attributable to this heterogeneity and to variability inherent in random field-based sampling. In addition, the absence of functional trophoblast assays limits definitive conclusions regarding the developmental equivalence to mature trophoblast lineages, warranting further investigation to define the extent of trophoblast maturation in this system.

FGF and BMP pathway inhibitors were used to investigate the requirements underlying the 2F-induced extraembryonic lineage. Consistent with previous porcine XEN studies (Zhang et al., 2021), which revealed that FGF2 inhibition compromises proliferation, FGF signaling inhibition in our system suggests that FGF activity is required for efficient establishment and proliferation of PrE/XEN-like cells. However, its effect on lineage-associated gene expression was more modest than that observed following BMP pathway inhibition. BMP signaling inhibition resulted in the downregulation of PrE-associated markers, including GATA4, GATA6, and SOX17, and also affected TE-associated markers, such as CDX2 and GATA3. These findings suggest that BMP signaling plays a central role in regulating lineage-associated gene expression under 2F conditions. Meanwhile, a recent study using porcine embryos reported that combined FGF2 and BMP4 treatment in N2B27 medium contributes to primitive endoderm formation during early embryonic development (Kim et al., 2026). In contrast, FGF2 and BMP4 treatment of pEPSCs under KOSR-containing N2B27 induction conditions activated PrE- and TE-associated transcriptional programs together with broader lineage-associated responses, rather than promoting a selectively stabilized PrE-like identity. These observations suggest that embryos, cell lines, and culture compositions may substantially influence developmental responses to FGF2 and BMP4 signaling. Importantly, our data do not support the conclusion that 2F treatment generates fully established XEN or trophoblast stem cell populations. Rather, the induced cells exhibit molecular features associated with extraembryonic lineages and represent a heterogeneous lineage-primed state.

In addition, FGF2 and/or BMP4 have previously been reported to induce mesoderm and TE lineages in PSCs (Yu et al., 2011; Drukker et al., 2012; Godoy-Parejo et al., 2023). Consistent with these reports, the bulk RNA-seq data in the present study indicate that 2F treatment induces extraembryonic lineage-associated programs while also upregulating the expression of mesoderm and ectoderm lineage markers. Notably, primitive streak markers (*TBXT, EOMES,* and *MIXL1*) did not show consistent upregulation in 2F-treated cells. *TBXT* exhibited modest and variable expression changes across biological replicates, whereas *EOMES* and *MIXL1* were not increased relative to pEPSCs. In contrast, other mesoderm-associated genes (*PAX2, ISL1, GATA5,* and *TBX6*) were upregulated. These findings suggest that 2F treatment promotes extraembryonic lineage-associated specification, although the induction process is accompanied by the upregulation of several mesoderm-associated genes. Importantly, these findings do not support robust activation of a canonical primitive streak transcriptional program under the current induction conditions. Furthermore, functional evidence for long-term self-renewal or stable lineage conversion was not obtained in the present study. Therefore, the induced populations are interpreted as extraembryonic lineage-associated priming states rather than definitive lineage-converted cells. Future studies involving long-term culture and serial passaging will be required to evaluate the stability and self-renewal potential of these induced populations. In addition, a limitation of the present study is that the transcriptomic analysis was confined to comparisons between pEPSCs and 2F-treated cells, without porcine embryos as reference controls. Therefore, further studies are required to determine the extent to which 2F-treated cells recapitulate native extraembryonic and other lineage states at the transcriptomic and functional levels.

A further interpretive challenge involves distinguishing PrE-like identity from definitive endoderm (DE), which arises from the primitive streak and has a distinct developmental origin from PrE (Nowotschin et al., 2019). DE and PrE share several transcription factors, including *GATA4*, *GATA6*, *SOX17*, and *HNF4A*, complicating lineage assignment based on these markers alone. In pigs, *GATA4* and *GATA6* are pan-endoderm markers whose expression patterns have been characterized in porcine embryos (Simpson et al., 2024). *SOX17* is a marker of both PrE and DE (Kanai-Azuma et al., 2002; Niakan et al., 2010; Simpson et al., 2024). 2F-treated cells expressed both general XEN markers and XEN-specific markers that distinguish them from DE. In particular, *NID2* and *HNF1B* have been identified as XEN-specific markers in both human and mouse models and were similarly upregulated in our dataset (Petropoulos et al., 2016; Linneberg-Agerholm et al., 2019). These findings support the interpretation that induced cells acquired PrE/XEN-like molecular features rather than representing a DE-like state.

In summary, this study demonstrates that a simple and defined combination of BMP4 and FGF2 (2F condition) induces PrE- and TE-associated molecular programs in pEPSCs, accompanied by acquisition of extraembryonic lineage-associated features. The 2F-treated cells also exhibited broader developmental plasticity. These results provide a useful framework for investigating early lineage transition dynamics, signaling-dependent cell-state remodeling, and the mechanisms underlying extraembryonic lineage priming in porcine stem cell models.

## Data Availability

The datasets presented in this study can be found in online repositories. The names of the repository/repositories and accession number(s) can be found below: https://www.ncbi.nlm.nih.gov/geo/, GSE319459.
