# Supplementary material for: BMP4 and FGF2 promote heterogeneous priming including extraembryonic lineages in porcine expanded potential stem cells
Source: Front Cell Dev Biol. 2026 Sep 9;14:1909585. doi: 10.3389/fcell.2026.1909585 (PMC13598362; doi:10.3389/fcell.2026.1909585)
Supplement: Supplementary file 1 [file Image1.pdf]

Supplementary Figure 1. SOX2 expression under different FGF2 and BMP4 concentrations.

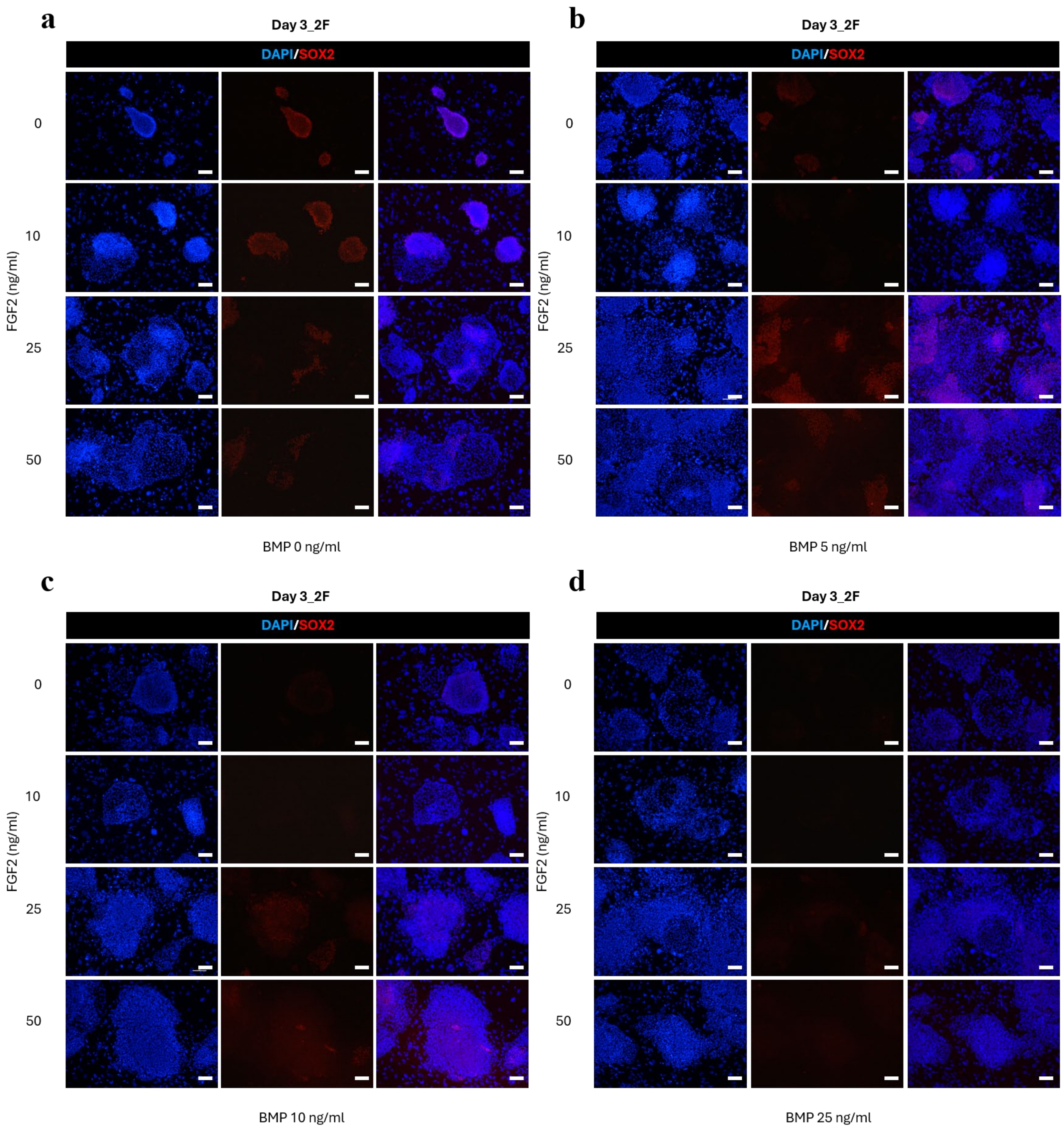

a-d) Representative immunofluorescence images of SOX2 expression following treatment with different combinations of FGF2 (0, 10, 25, and 50 ng/mL) and BMP4 (0, 5, 10, and 25 ng/mL) for 3 days. Scale bar, 100 μm.

Supplementary Figure 2. HNF4A and CDX2 expression under different FGF2 and BMP4 concentrations.

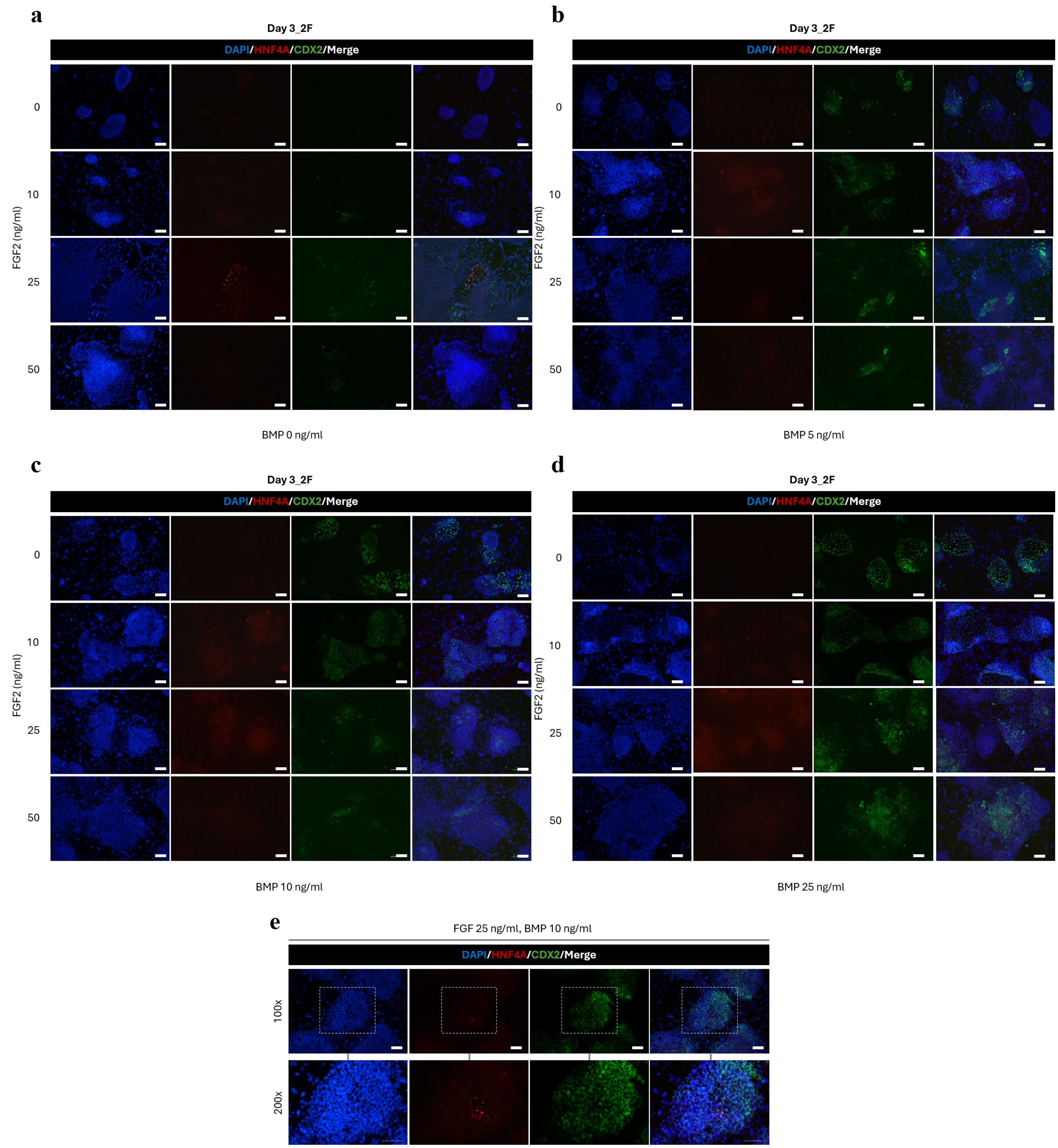

a-d) Representative immunofluorescence images of HNF4A and CDX2 expression following treatment with different combinations of FGF2 (0, 10, 25, and 50 ng/mL) and BMP4 (0, 5, 10, and 25 ng/mL) for 3 days. Scale bar, 100 µm. e) Additional representative immunofluorescence images showing HNF4A and CDX2 expression in the FGF2 (25 ng/mL)/BMP4 (10 ng/mL) condition at 100× and 200× magnification, illustrating the field-to-field variation in lineage-marker expression. Scale bars, 100 µm (100× and 200×). The white dashed box indicates the magnified region.

Supplementary Figure 3. Quantification of lineage marker expression across FGF2/BMP4 treatment conditions.

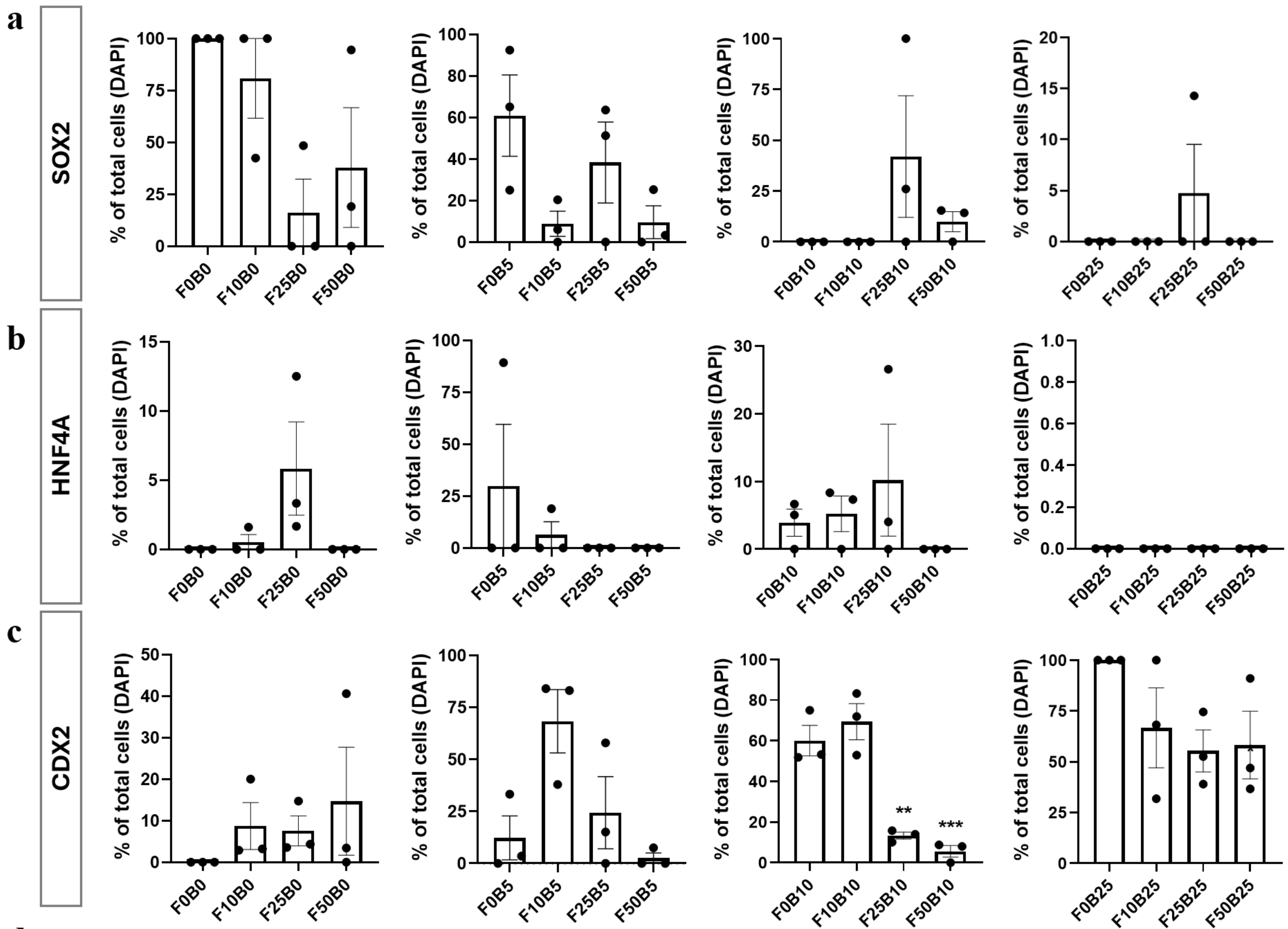

d

SOX2 +					HNF4A +					CDX2 +				
FGF2 (ng/mL)	BMP4 0	BMP4 5	BMP4 10	BMP4 25	FGF2 (ng/mL)	BMP4 0	BMP4 5	BMP4 10	BMP4 25	FGF2 (ng/mL)	BMP4 0	BMP4 5	BMP4 10	BMP4 25
0	100.00 ± 0.00	60.85 ± 19.61	0.00 ± 0.00	0.00 ± 0.00	0	0.00 ± 0.00	29.76 ± 29.76	3.91 ± 2.01	0.00 ± 0.00	0	0.00 ± 0.00	12.30 ± 10.57	60.08 ± 7.47	100.00 ± 0.00
10	80.81 ± 19.19	8.80 ± 6.06	0.00 ± 0.00	0.00 ± 0.00	10	0.54 ± 0.54	6.32 ± 6.32	5.23 ± 2.63	0.00 ± 0.00	10	8.71 ± 5.65	68.36 ± 15.22	69.41 ± 8.87	66.70 ± 19.67
25	16.15 ± 16.15%	38.30 ± 19.72	*42.00 ± 30.82	4.76 ± 4.76	25	5.83 ± 3.37	0.00 ± 0.00	*10.20 ± 8.26	0.00 ± 0.00	25	21.53 ± 10.36	24.37 ± 17.36	*13.32 ± 1.68	55.39 ± 10.35
50	37.87 ± 27.93%	9.53 ± 7.82	9.91 ± 5.03	0.00 ± 0.00	50	0.00 ± 0.00	0.00 ± 0.00	0.00 ± 0.00	0.00 ± 0.00	50	14.68 ± 13.00	2.52 ± 2.52	5.62 ± 2.82	58.23 ± 16.65

e

25 FGF2/10 BMP4 ng/ml		
n = 3 fields	HNF4A + (%)	CDX2 + (%)
1	0	62.03
2	10.81	77.03
3	0	88.46
Mean ± SEM	3.60 ± 3.60	75.84 ± 7.65

Immunofluorescence quantification of the percentage of $DAPI^+$ cells positive for a) SOX2 (pluripotency marker), b) HNF4A (hypoblast/hepatic marker), and c) CDX2 (trophectoderm/extraembryonic marker) following treatment with the indicated combinations of FGF2 (0, 10, 25, or 50 ng/mL) and BMP4 (0, 5, 10, or 25 ng/mL). P values were calculated using one-way ANOVA. * $p < 0.05$, **** $p < 0.0001$. d) Summary table of the percentage of $SOX2^+$, $HNF4A^+$, and $CDX2^+$ cells shown in (a-c), presented as mean ± SEM for each FGF2/BMP4 combination. Asterisk indicates the FGF2 25 ng/mL and BMP4 10 ng/mL condition. e) Quantification of the percentages of $HNF4A^+$ and $CDX2^+$ cells among $DAPI^+$ cells in the additional representative immunofluorescence images for the FGF2 (25 ng/mL)/BMP4 (10 ng/mL) condition shown in Supplementary Fig. 2e. Data represent mean ± SEM from three randomly selected fields per sample (n = 3).

This is a provisional file, not the final typeset article

Supplementary Figure 4. Individual and combined effects of FGF2 and BMP4 on SOX2 and CDX2 expression in pEPSCs.

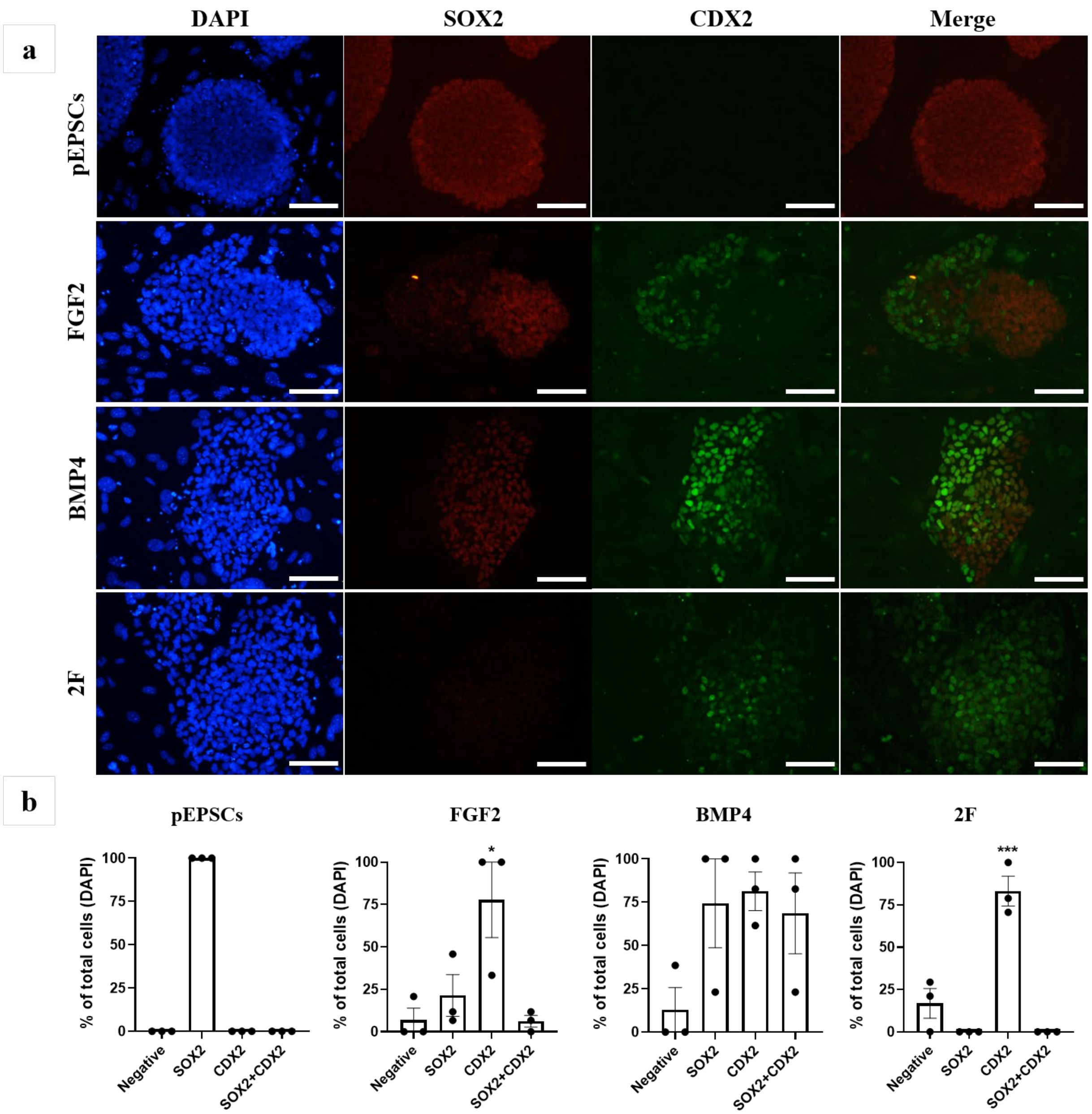

Condition	Negative	SOX2	CDX2	MERGE (SOX2+CDX2)
pEPSCs	0.0±0.0	100.0±0.0	0.0±0.0	0.0±0.0
FGF2	6.9±6.9	21.4±12.3	77.8±22.2	6.2±3.5
BMP4	12.8±12.8	74.4±25.6	81.4±11.2	68.6±23.5
2F	16.8±8.8	0.0±0.0	83.2±8.8	0.0±0.0

a) Immunofluorescence analysis of pluripotency (SOX2) and TE (CDX2) markers in pEPSCs following treatment with FGF2 alone, BMP4 alone, or both factors (2F) on day 3. Scale bar, 100 µm. **b)** Quantification of the proportion of Negative, $SOX2^+$, $CDX2^+$, and $SOX2^+CDX2^+$ (co-expressing) cells relative to total cells ($DAPI^+$) under each treatment condition. Quantification was performed on three randomly selected microscopic fields from the same sample and is presented as mean ± SEM (n = 3). P values were calculated using one-way ANOVA. *$p < 0.05$, ***$p < 0.001$.

Supplementary Figure 5. RT-qPCR validation of primitive Streak, mesoderm, and ectoderm marker induction in 2F-treated cells.

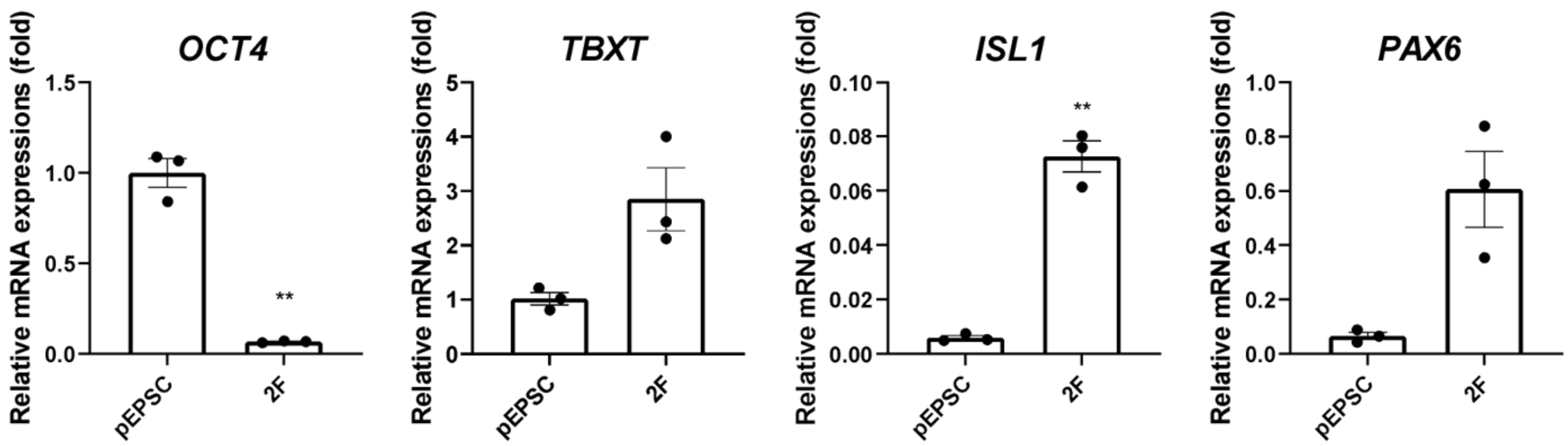

RT-qPCR analysis of the expression of pluripotency (*OCT4*), primitive streak (*TBXT*), mesoderm (*ISL1*), and ectoderm (*PAX6*) markers in 2F-treated cells. 2F, combined FGF2 and BMP4. Data represent the mean ± SEM(n = 3). mRNA expression levels were quantified by qPCR and normalized to GAPDH. The p-values were calculated using Welch's t-test. ** $p < 0.01$.
