# Supplementary material for: BMP4 and FGF2 promote heterogeneous priming including extraembryonic lineages in porcine expanded potential stem cells
Source: Front Cell Dev Biol. 2026 Sep 9;14:1909585. doi: 10.3389/fcell.2026.1909585 (PMC13598362; doi:10.3389/fcell.2026.1909585)
Supplement: Supplementary file 2 [file Table1.docx]

**Supplementary Table 1** List of primers used for gene expression analysis

| Genes | | 5′-Primer sequences-3′ | | Product size (bp) | | Gene Bank  accession number | Ref. | |
| --- | --- | --- | --- | --- | --- | --- | --- | --- |
| *GAPDH* | F: 5′-CAGGTTGTGTCCTGTGACT-3′ | | 102 | | NM_001206359.1 | | |  |
|  | R: 5′-CCAGGAAATGAGCTTGACGA-3′ | |  |  |  |  |  |  |
| *POU5F1* | F: 5’-CCAAGCTCCTAAAGCAGAAGAG-3’ | | 106 | | NM_001113060.1 | | |  |
|  | R: 5’-CAGATGGTCGTTTGGCTGAA-3’ | |  |  |  |  |  |  |
| *SOX2* | F: 5’-AACAGCCCAGACCGAGTTAAG-3’ | | 254 | | NM_001123197.1 | | | Kim et al., 2023 |
|  | R: 5’-ATGAGCGTCTTGGTTTTCCG-3’ | |  |  |  |  |  |  |
| *GATA3* | F: 5’-GGACAGAATCGACCCCTTAT-3’ | | 197 | | NM_001044567.1 | | | Kim et al., 2023 |
|  | R: 5’-CCTTCCTTCTTCATGGTCAG-3’ | |  |  |  |  |  |  |
| *CDX2* | F: 5’-CTGTTTGGGTTGTTGGTCTG-3’ | | 95 | | NM_001278769.1 | | | Kim et al., 2023 |
|  | R: 5’-CCCACTCCCTTCACCATATC-3’ | |  |  |  |  |  |  |
| *GATA4* | F: 5′-CGACACCCTAATCTCGATATGTT-3’ | | 143 | | NM_214293.1 | | | Li et al., 2020 |
|  | R: 5′-TTCATCTTGTGGTAGAGGCCG-3’ | |  |  |  |  |  |  |
| *GATA6* | F: 5′-TCGTTTGGTACACACCTCTG-3’ | | 147 | | NM_214328.2 | | |  |
|  | R: 5′-ACTTCGGGTCAGTCACACA-3’ | |  |  |  |  |  |  |
| *SOX17* | F: 5′-TGGTTGAATCTTGAGGTCTGC-3’ | | 119 | | XM_001928376.7 | | |  |
|  | R: 5′-CAGGGTGTAGGTGTGTGATG-3’ | |  |  |  |  |  |  |
| *HNF4A* | F: 5’-GGTCAAGCTACGAGGATAGC-3’ | | 209 | | NM_001044571.1 | | |  |
|  | R: 5’-CTCACAGAAAGCTGGGATGT-3’ | |  |  |  |  |  |  |
| *FGFR1* | F: 5’- TCTTCTCTCACGAACCCTTG | | 126 | | XM_005671767.3 | | |  |
|  | R: 5’-AGCTAAGGATGGAGTTTGGG | |  |  |  |  |  |  |
| *FGFR2* | F: 5’- GTAACAGTTTCTGCCGAGTC | | 167 | | NM_001099924.3 | | |  |
|  | R: 5’- AGCGTCAGCTTATCTCTGG | |  |  |  |  |  |  |
| *BMPR1A* | F: 5’- ATGGTTCGGCAAGTTGGTA | | 147 | | NM_001204767.1 | | |  |
|  | R: 5’- GCGCATTAGCACAGTTTGATAG | |  |  |  |  |  |  |
| *SMAD1* | F: 5’- GAGGAACCCAAACACTGGT | | 165 | | NM_213965.1 | | |  |
|  | R: 5’- ATTCCGGTTGACGTTGGA | |  |  |  |  |  |  |
| *SMAD5* | F: 5’- CGGTGGCTCGATGAATCAAT | | 149 | | XM_021084786.1 | | |  |
|  | R: 5’- AAGTGGCTCAAACCAGGTC | |  |  |  |  |  |  |

F: Forward, R: Reverse

**Supplementary Table 2 List of antibodies used for immunofluorescence staining**

| Detection of | Name | Host species | Dilution or Concentration | Manufacturer (Cat #) |
| --- | --- | --- | --- | --- |
| Pluripotency | SOX2 | Mouse / IgG | 1:400 | Cell Signaling  (#4900) |
| TE | CDX2 | Rabbit / IgG | 1:200 | Cell Signaling  (#12306) |
| PrE | SOX17 | Rabbit / IgG | 1:3200 | Cell Signaling  (#81778) |
| PrE | HNF4A | Mouse / IgG | 1:1200 | Abcam  (ab41898) |
| 2’ antibody | Alexa Fluor 488 | Donkey / IgG | 2 µg/mL or 1:200 | Invitrogen  (A21206) |
| 2’ antibody | Alexa Fluor 594 | Donkey / IgG | 4 µg/mL or 1:200 | Invitrogen  (A21203) |

Supplementary Table 3 Amino acid sequence identity between human and porcine FGF2, FGF4, and BMP4.

| Gene | Species | Accession | Length (aa) | Query Cover (%) | Percent identity (%) | Positives (%) |
| --- | --- | --- | --- | --- | --- | --- |
| FGF2 | Pig | NP_001392443.1 | 155 | 100 | 98.71 | 99.35 |
|  | Human | NP_001348594.1 | 155 |  |  |  |
| FGF4 | Pig | XP_003122466.1 | 206 | 100 | 92.23 | 94.66 |
|  | Human | NP_001998.1 | 206 |  |  |  |
| BMP4 | Pig | NP_001094501.1 | 409 | 100 | 98.29 | 98.78 |
|  | Human | NP_001334843.1 | 408 |  |  |  |

Percent identity and similarity were determined by pairwise NCBI BLASTp alignment (Blast 2 sequences) of full-length RefSeq protein sequences for *Homo sapiens* and *Sus scrofa.*
